# Extracellular vesicles carrying surface-anchored adiponectin prevent obesity-related metabolic complications by enhancing insulin sensitivity

**DOI:** 10.1016/j.molmet.2026.102361

**Published:** 2026-04-01

**Authors:** Alexia Blandin, Margot Voisin, Josy Froger, Maëlle Lachat, Grégory Hilairet, Katarzyna Polak, Julie Magusto, Lisa Meslier, Mikaël Croyal, Mathilde Gourdel, Quentin Massiquot, Julien Chaigneau, Maxime Carpentier, Lionel Fizanne, Xavier Prieur, Cédric Le May, Jérôme Boursier, Bertrand Cariou, Robert Mamoun, Bernadette Trentin, Soazig Le Lay

**Affiliations:** 1 Nantes Université, CHU Nantes, CNRS, INSERM, l'institut du thorax, Nantes, F-44000, France; 2 Université Angers, SFR ICAT, Angers, 49000, France; 3 Ciloa, 356 rue Maurice Béjart, Montpellier, 34080, France; 4 Nantes Université, CHU Nantes, Inserm, CNRS, SFR Santé, Inserm UMS 016, CNRS UMS 3556, Nantes, France; 5 HIFIH, CHU Angers, Université Angers, SFR ICAT, Angers, 49000, France

**Keywords:** Extracellular vesicles, Exosomes, Obesity, Type 2 diabetes, MASLD, MASH, Bioengineering

## Abstract

Adiponectin (Adpn) is a potent insulin-sensitizing adipokine with therapeutic promise for type 2 diabetes (T2D) and metabolic dysfunction-associated steatohepatitis (MASH). Its clinical use is limited by challenges in producing stable, bioactive high-molecular weight forms. Adipocyte-derived extracellular vesicles (EVs) naturally carry oligomeric Adpn on their surface, enhancing hormone stability and activity. Here, we engineered EVs displaying membrane-anchored Adpn (EV^PP−Adpn^) and control EVs lacking Adpn (EV^CTL^), and evaluated their metabolic effects in high fat diet (HFD)-induced obesity mice.

EV^PP−Adpn^ were purified from HEK293T cells stably transfected with a chimeric Adpn fused to a transmembrane domain and a pilot peptide (PP) directing it to EVs; EV^CTL^ were produced from non-transfected cells. HFD-fed male and female mice received intraperitoneal EV injections for six weeks.

EV^PP−Adpn^ improved glucose tolerance and insulin sensitivity, promoted adipocyte lipid storage through insulin-regulated lipogenesis and alleviated MASH features (liver steatosis, inflammation and fibrosis). EV^PP−Adpn^ lowered circulating ceramides and reduced FGF21, indicating improved hepatic metabolism, and activated AKT and AMPK pathways in liver and skeletal muscle, consistent with increased adiponectin signaling.

These results demonstrate that surface-anchored Adpn EVs restore tissue-specific insulin signaling and improve obesity-related metabolic dysfunctions, highlighting their potential as a novel biotherapeutic strategy for T2D and MASH.

## Highlights

• EV^PP−Adpn^ are engineered EVs displaying surface-anchored adiponectin (Adpn) oligomers.
• EV^PP−Adpn^ improve glucose tolerance and insulin sensitivity in obese mice.
• EV^PP−Adpn^ alleviate MASH features like steatosis, inflammation, and fibrosis.
• EV^PP−Adpn^ activate AKT and AMPK signaling pathways in metabolic tissues.
• Adpn-exposing EVs act as promising therapeutic platforms for managing T2D and MASH.

## 1 Introduction

The rising prevalence of type 2 diabetes (T2D) and its associated cardiometabolic complications underscores the need for innovative therapeutic strategies, especially those aimed at restoring insulin sensitivity. Despite major advances in glucose-lowering therapies, there remains a critical lack of disease-modifying approaches capable of simultaneously improving systemic insulin resistance and associated metabolic organ dysfunctions, including metabolic dysfunction-associated steatohepatitis (MASH).

Adiponectin (Adpn), an adipokine with insulin-sensitizing, anti-inflammatory, and cardioprotective properties is an attractive therapeutic target [1]. High-molecular-weight (HMW) oligomers are recognized as the biologically active forms of the hormone [2,3]. Adpn oligomers signal through AdipoR1/2 to activate AMPK and PPARa-dependent metabolic pathways [4]. AdipoR signaling also cross-talks with the insulin receptor and its downstream PI3K/AKT signaling pathways, thereby enhancing insulin [5,6]. As a key transcriptional target of PPARγ, Adpn moreover accounts for many of the primary metabolic effects of PPARγ-activating agents such as thiazolidinediones (TZDs) [7,8] and FGF21 [9]. However, clinical use of recombinant Adpn has been limited by the difficulties encountered in producing stable HMW oligomers, which require complex post-translational modifications that are technically challenging to reproduce [10].

EVs are lipid bilayer-enclosed structures that transport bioactive molecules, including proteins, lipids, and nucleic acids [11,12]. Their high bioavailability, biocompatibility, and low immunogenicity make them promising therapeutic delivery nanocarriers [12,13]. Beyond their role as passive carriers, EVs offer a unique opportunity to present membrane-associated ligands in a spatially organized and biologically relevant configuration, a feature that is difficult to achieve with recombinant proteins [12,14].

Adipocyte-derived extracellular vesicles (EVs) naturally carry multimeric Adpn on their surface [15,16]. We previously showed that this EV-associated Adpn accounts for ∼20% of circulating Adpn and exhibits higher stability in the bloodstream than free Adpn, while retaining the insulin-sensitizing properties of the adipokine through AdipoR signaling [15].

Here, we develop a bioengineered EV-based strategy that uses EVs as delivery vehicles for bioactive Adpn oligomers, paving the way for their use as a therapeutic platform. To this goal, we engineered EVs presenting anchored oligomerized Adpn forms on their surface (EV^PP−Adpn^) to target metabolic tissues and evaluate their potential in mitigating insulin resistance and metabolic dysfunctions in high-fat diet (HFD)-fed mice. Six weeks of EV^PP−Adpn^ treatment improve insulin sensitivity, promote adipocyte lipid storage through insulin-regulated lipogenesis and alleviate key features of MASH lesions (steatosis, inflammation, fibrosis) by restoring hepatic sensitivity. Altogether, our results highlight that surface Adpn-carrying EVs as a promising biotherapy for managing T2D and MASH.

## 2 Results

### 2.1 Generation and characterization of bioengineered EVs carrying Adpn.

To exploit the therapeutic potential of surface Adpn-associated EVs, we engineered EVs carrying native membrane-anchored Adpn using a patented technology (see ESM methods). A chimeric construct (PP-Adpn) fused Adpn to a transmembrane domain (TM) and a pilot peptide (PP) that directs it to the EV secretion pathway (Fig. S1). EV^PP−Adpn^ were isolated from the supernatants of stably transfected PP-Adpn-HEK 293T cells, while control EVs (EV^CTL^) were obtained from non-transfected cells (see Supplementary material). Both EV preparations were enriched in EV markers Alix, syntenin-1 and CD81, exhibited similar size distributions with a mean diameter of ∼70 nm and yielded EV concentrations of approximately ∼10^10^-10^11^ particles/mL (Fig. S1B–F). Adpn was consistently loaded in bioengineered EV^PP−Adpn^ batches, absent in EV^CTL^, and detected as high-molecular-weight, metabolically active forms (Fig. S1G–H).

### 2.2 Surface Adpn-anchored EVs (EV^PP−Adpn^) improve insulin sensitivity

**Figure 1:**
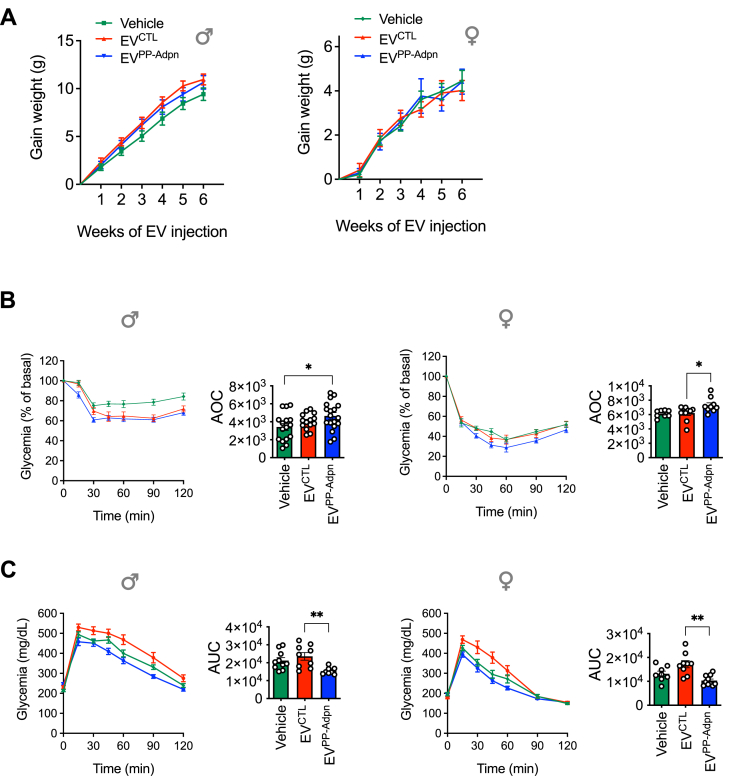
**Surface-anchored Adpn-EVs (EV^PP−Adpn^) improve insulin sensitivity and glucose tolerance in HFD-fed mice**. **(A)** Weight gain trajectory male (left) and female (right) HFD-fed mice after 6 weeks of i.p. injections with EV^CTL^ and EV^PP−Adpn^ or vehicle (PBS). **(B)** Insulin tolerance tests (ITT) performed in males (left) and females (right), with area over the curve (AOC) presented on the right. **(C)** Intraperitoneal glucose tolerance tests (GTT) in males (left) and females (right), with area under the curve (AUC) shown on the right.

Male and female HFD-fed mice were injected twice weekly for 6 weeks with Vehicle (PBS), EV^CTL^ or EV^PP−^Adpn to assess their metabolic effects, following our EV-injection protocol that previously revealed the beneficial effects of vesicular Adpn from adipocyte-derived EVs [15]. No significant differences in body weight gain over time (Figure 1A) or tissue weights (adipose depots, liver, pancreas, spleen and muscle; Fig. S2A) were observed. Blood biochemistry analyses (Table S1) showed no signs of hepatic or or renal toxicity, with even a slight trend toward lower transaminase levels in HFD-fed mice injected with EV^CTL^ compared to Vehicle group, supporting the absence of toxicity and confirming the good tolerance of repeated EV injections.

Although random-fed and fasting glycemia remained stable in both sexes (Fig. S2B–C), EV^PP−Adpn^ significantly improved insulin sensitivity in males and females compared to Vehicle, as shown by increased area over the curve in ITT (Figure 1B). IP-GTT further demonstrated enhanced glucose tolerance in EV^PP−Adpn^-treated mice versus EV^CTL^ (Figure 1C). In males, this effect was accompanied by restoration of the 15-min insulin peak after glucose load, absent in EV^CTL^, Vehicle-treated groups, and all females (Fig. S2D). These benefits occurred without changes in pancreatic β-cell mass or size, as confirmed by comparable insulin immunostaining across groups (Fig. S2E–H).

### 2.3 EV^PP−Adpn^ promote healthy adipose tissue remodeling

**Figure 2:**
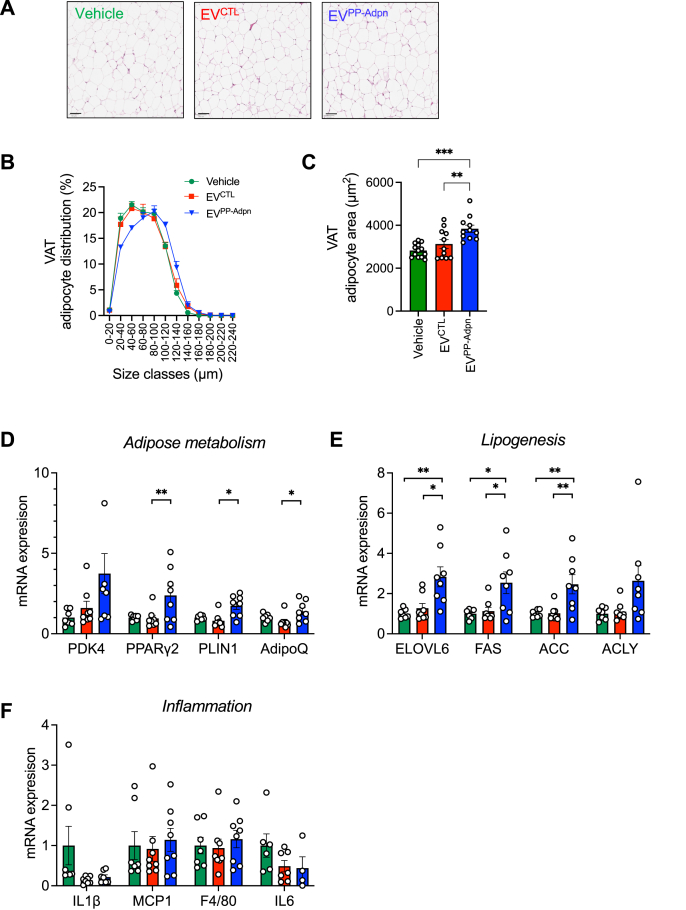
**EV^PP−Adpn^ promote healthy adipose tissue remodeling**. **(A**–**C)** Histological analysis of VAT in male HFD-fed mice at sacrifice. Representative hematoxylin/eosin-stained VAT sections are shown **(A)**, from which adipocyte diameters were measured. Analysis of VAT adipocyte size distribution is presented in **(B)**, and mean adipocyte area in **(C)**. n = 11–12 males per group were analyzed. Scale bar, 100 μm. **(D**–**F)** Quantitive PCR gene expression analysis in VAT from male HFD-fed injected mice. mRNA expression was analyzed for the key adipogenic markers PDK4, PPARγ2, PLIN1, AdipoQ **(D)**, for the insulin-responsive lipogenic genes ELOVL6, FAS, ACC, ACLY **(E)** and for the pro-inflammatory markers IL1β, MCP1, F4/80, IL6 **(F)**. Data are presented as mean ± SEM. Dot plots represent the number of independent animals analyzed. Statistical differences were calculated using one-way ANOVA followed a Tukey's multiple comparisons test. ∗p < 0.05, ∗∗p < 0.01. Bar colors: Green, Vehicle; Red, EV^CTL^; Blue, EV^PP−Adpn^.

Although total AT mass was unchanged (Fig. S2A), visceral adipose tissue (VAT) histology showed larger adipocytes in EV^PP−Adpn^ groups (Figure 2A–C), an effect not seen in subcutaneous adipose tissue (SAT, Fig. S2I–J). EV^PP−Adpn^ increased key adipocyte markers (*PPARγ2, PLIN1, AdipoQ*) and insulin-regulated lipogenic enzymes (*ELOVL6, FAS, ACC, ACLY*) without affecting inflammatory gene expression (Figure 2D–F). Circulating non-esterified fatty acids (NEFA) levels were unchanged (Table S1), indicating that basal lipolysis was unaffected. Together, these results show that surface Adpn-associated EVs promote insulin-sensitive VAT expansion and healthy adipose tissue remodeling.

### 2.4 EV^PP−Adpn^ alleviate liver dysfunction and improve key metabolic signaling pathways

**Figure 3:**
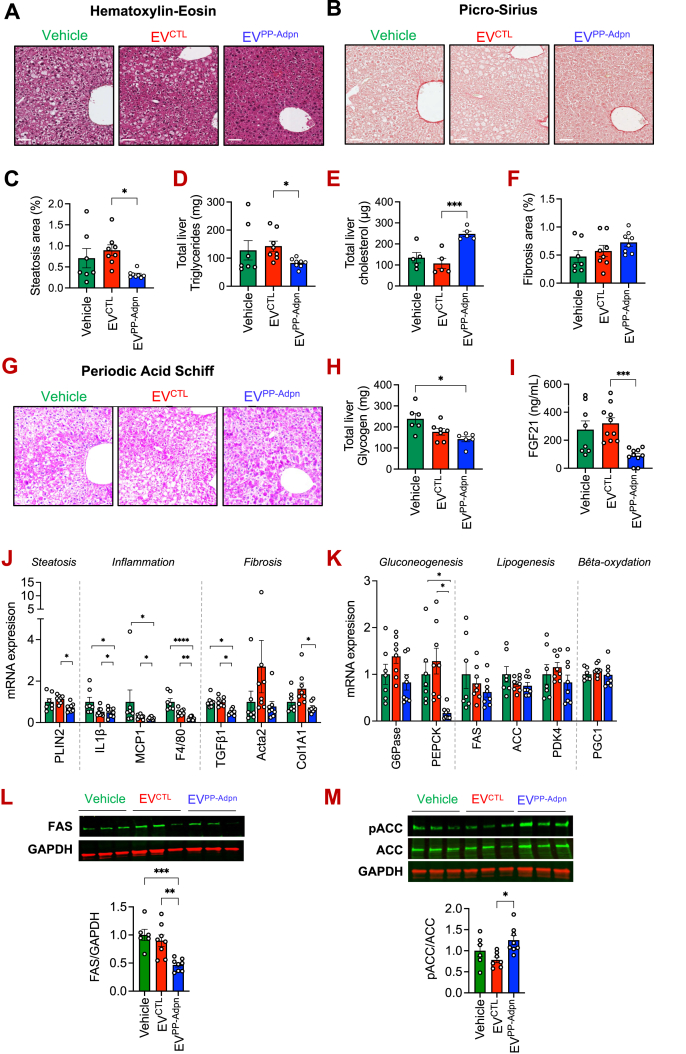
**EV^PP−Adpn^ alleviate liver dysfunction and improve key metabolic signaling pathways**. **(A**–**H)** Histological analysis of liver sections. Representative hematoxylin–eosin **(A)** and picrosirius red **(B)** and periodic acid-Schiff **(G)** staining are shown for male mice after 6 weeks of treatment with EV^CTL^ or EV^PP−Adpn^ or vehicle (PBS). Quantification of steatosis area on HE-stained sections **(C)** and fibrosis area (%) on PS-stained sections (F) are presented. Total liver content of cholesterol **(D)**, triglycerides**(E)**,glycogen **(H)** have been quantified. n = 5–8 animals per group were analyzed. Scale bar, 100 μm. **(I)** Circulating FGF-21 in injected male mice. **(J**–**K)** Quantitative PCR analysis of hepatic gene expression in HFD-fed injected male mice. Relative mRNA levels of MASLD-associated genes **(G)** including steatotic (PLIN2), pro-inflammatory (IL1β, MCP1, F4/80) and fibrotic markers (TGFβ1, Col1A1). Expression of key metabolic genes **(H)** related to gluconeogenesis (PEPCK, G6Pase), lipogenesis (FAS, ACC) and β-oxidation (PDK4, PGC1α). **(L**–**M)** Western blot analysis of FAS **(L)** and ACC (Ser79) phosphorylation **(M)** in random-fed mice liver samples. Representative blots are shown, with GAPDH as loading control. Quantification of FAS/GAPDH **(L)** and phospho-ACC/total ACC ratios **(M)** is presented below the blots.

Liver histology revealed a marked reduction in steatosis in EV^PP−Adpn^–treated males, as highlighted by a significant decrease in hepatic triglyceride content compared to EV^CTL^ injected animals (Figure 3A; 3C-D). Conversely, hepatic total cholesterol was two-fold higher in EV^PP−Adpn^ treated mice compared with EV^CTL^ (Figure 3E). Importantly, circulating total cholesterol and triglyceride levels were unchanged between mice groups (Table S1), suggesting that the observed hepatic cholesterol increase does not reflect major systemic lipid alterations. Liver fibrosis, already minimal in HFD-fed animals, remained unchanged (Figure 3B; 3F). Hepatic glycogen was also evaluated by PAS staining (Figure 3G), which showed no apparent differences among Vehicle, EV^CTL^ and EV^PP−Adpn^ groups. However, quantitative measurements revealed a significant decreased in total liver glycogen in EV^PP−Adpn^ treated mice (Figure 3H), which may contribute to the observed marked liver histological changes observed in these livers (Figure 3A). Liver enzymes (ALT, AST) were significantly reduced in EV^PP−Adpn^ treated mice compared to Vehicle, whereas the modest decrease observed following EV^CTL^ remained non-significant (Table S1). In contrast, EV^PP−Adpn^ markedly lowered circulating FGF21 compared with both EV^CTL^ and Vehicle, highlighting a specific Adpn-associated EV effect (Figure 3I). As circulating Fibroblast Growth Factor 21 (FGF21) and liver transaminases are typically elevated under HFD-induced metabolic stress, their coordinated reduction is consistent with improved hepatic function.

At the transcriptional level, EV^PP−Adpn^ downregulated metabolic dysfunction–associated steatotic liver disease (MASLD)-related genes including the lipid droplet marker *PLIN2*, and pro-inflammatory (IL1β, MCP1, F4/80) and fibrotic markers (TGFβ1, Col1A1) (Figure 3J). Although most hepatic metabolic genes remained unchanged, EV^PP−Adpn^ restored insulin-mediated repression of PEPCK, with a similar trend for G6Pase (Figure 3K), likely limiting glucose-6-phosphate availability for glycogen synthesis (Figure 3H). At the protein level, immunoblots revealed a significant reduction of the lipogenic enzyme FAS in EV^PP−Adpn^ treated livers (Figure 3L), accompanied by increased phosphorylation of ACC (Figure 3M), which would reduce malonyl-CoA production and limit *de novo* fatty acid synthesis.

Overall, these findings indicate that EV^PP−Adpn^ improve hepatic insulin sensitivity and alleviates key features of MASH.

### 2.5 EV^PP−Adpn^ improve tissue-specific insulin signaling via activation of the adiponectin pathway

**Figure 4:**
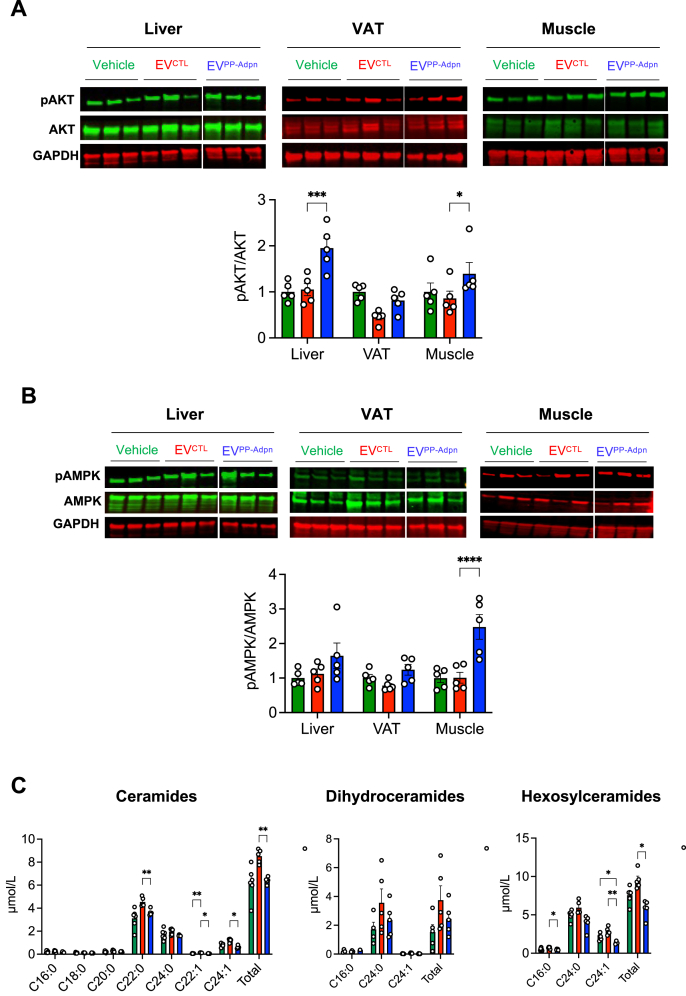
***EV*^*PP*-*Adpn*^*improve tissue-specific insulin signaling* via *activation of the adiponectin pathway***. **(A**–**B)** Western blot analysis of AKT (Ser473) **(A)** and AMPK (Thr172) **(B)** phosphorylation in liver, VAT, and skeletal muscle following insulin injection in mice. Representative blots are shown, with GAPDH as loading control. Since EV^PP−Adpn^ and EV^CTL^ samples were run on the same gel but not in adjacent lanes, the non-adjacent lanes were cropped for clarity. Quantification of phospho-AKT/total AKT **(B)** and phospho-AMPK/total AMPK ratios **(B)** is presented below the blots. **(C)** Plasma ceramide profiling showing total levels and subspecies of ceramides (left panel), dihydroceramides (middle panel) and hexosylceramides (right panel). Data are presented as mean ± SEM. Dot plots represent the number of independent animals analyzed. Bar colors: Green, Vehicle; Red, EV^CTL^; Blue, EV^PP−Adpn^.

We next assessed adiponectin-responsive signaling in VAT, liver and skeletal muscle of male mice. Insulin-stimulated AKT (Ser473) phosphorylation — a readout of insulin sensitivity — was increased in all tissues of EV^PP−Adpn^ treated mice compared to EV^CTL^ mice, with significant effects in liver and muscle (Figure 4A). Given the central role of AMPK in adiponectin-driven regulation of lipid metabolism and energy homeostasis, we assessed AMPK (Thr172) phosphorylation. EV^PP−Adpn^ promoted a general increase across tissues, reaching significance in muscle, indicative of improved metabolic function (Figure 4B).

Because surface-anchored Adpn on EV^PP−Adpn^ is expected to activate AdipoR signaling [15], which promotes ceramidase activity, we measured plasma ceramides. EV^PP−Adpn^ treatment significantly decreased total ceramides and hexosyl-ceramides, particularly long-chain subspecies (C22:0 to C24:0) (Figure 4C). Altogether, these findings support that EV^PP−Adpn^ enhance tissue-specific insulin signaling through activation of the adiponectin pathway, leading to metabolic improvements at both the systemic and organ levels.

## 3 Discussion

EV^PP−Adpn^, engineered to display oligomeric adiponectin anchored at the vesicle surface, demonstrated insulin-sensitizing effects in HFD-fed mice, promoting adipocyte lipid storage and alleviating liver steatosis. The reduced expression of pro-inflammatory and fibrotic genes following Adpn-enriched EV treatment further supports an improvement in hepatic inflammation. However, these effects may be limited by the relatively mild liver challenge induced by the HFD used in this study compared with more aggressive MASH-inducing diets such as high-fat/high-sucrose/high-cholesterol regimens. Nevertheless, these findings suggest that EV^PP−Adpn^ may exert hepatoprotective effects beyond liver steatosis. While the precise mechanism was not directly assessed in this study, anchoring of Adpn at the vesicle surface likely promotes AdipoR-mediated signaling, as suggested by our previous results with adipose-derived EVs [15]. Although EVP^P−Adpn^ improve insulin sensitivity in both male and female mice, metabolic responses differ slightly as illustrated by the lack of an insulin secretion peak after a glucose challenge in females. These sex-specific differences may reflect distinct regulation of adiponectin signaling, as females have higher circulating adiponectin levels, potentially further enhanced by estrogen effects [17]. The activation of the PPARγ pathway in VAT, the improvement in hepatic insulin sensitivity, and the activation of AMPK in skeletal muscle collectively demonstrate that the EV^PP−Adpn^ efficiently activate adiponectin signaling across these target tissues. At the hepatic level, EV^PP−Adpn^ treatment is associated with a significant reduction of FAS protein and increased phosphorylation of ACC, suggesting that suppression of *de novo* lipogenesis may contribute to the observed decrease in liver triglyceride content. As a consequence, the promotion of adipocyte lipid storage is consistent with enhanced insulin sensitivity and resembles the effects of other insulin-sensitizing agents, such as thiazolidinediones, whose metabolic actions have also been linked to adiponectin signaling [18].

Despite these benefits, EV^PP−Adpn^ appear somewhat less potent than native adipose EVs from our previous studies under comparable experimental conditions [15], suggesting that additional vesicular cargo may act synergistically to further enhance insulin sensitivity [19]. This concept is supported by several recent studies highlighting the therapeutic potential of engineered EVs with adipocyte-derived metabolic active molecules: leptin-loaded macrophage-derived EVs sucessfully targeted the brain and restored breathing function in obese mice, overcoming leptin resistance [20]. Similarly to our bioengineered Adpn-enriched EVs, apelin-loaded EVs improved glucose tolerance and activated AMPK and Akt pathways in diabetic mice, enhancing systemic insulin sensitivity [21]. Finally, EVs combining surface FGF21 with miR-223 achieved liver-preferential delivery and reduced the MASH phenotype [22]. These examples highlight the potential of combinatorial cargo strategies to further potentiate EV^PP−Adpn^ and optimize therapeutic efficacy in insulin resistance and metabolic diseases.

## 4 Methods

### 4.1 Animal experimentation

Male and female C57BL/6J (B6) mice (10 weeks old) were fed a HFD (D12492, Safe Diets) *ad libitum* for 6 weeks and housed under standard conditions. Mice received biweekly intraperitoneal injections of EVs (EV^CTRL^ lacking Adpn or EV^PP−Adpn^) resuspended in 100 μL sterile 0.9% NaCl, or an equivalent volume of Vehicle (PBS in 0.9% NaCl). A 25 ng Adpn-equivalent EV dose, or the corresponding particle numbers for EV^CTL^, was selected based of our previous work, in which 5 μg injected adipose-derived EVs delivered ∼5 ng Adpn and ∼5 × 10^9^ particles per injection as measured by NTA (ZetaView) [15]. In the present study, the 25 ng Adpn-equivalent dose corresponds to ∼5–6 × 10^8^ particles by NanoFCM and ∼1 × 10^9^ by NTA, i.e. a particle number in the same order of magnitude but carrying a higher Adpn load (see Fig. S1I).

During the fifth week of EV injections, glucose (GTT) and insulin (ITT) tolerance tests were performed after a 4 h of fasting. For GTT, mice received 1.25 g/kg glucose i.p., and for ITT, 0.75 IU/kg human insulin (Humulin). Blood glucose was measured from tail vein samples up to 120 min (Accu-Chek Guide).

At sacrifice, body and tissue weights were recorded in random-fed conditions or after a 4 h fast followed by insulin injection (3 IU/kg; Humulin**,** Lilly). Tissues were fixed for histology or snap-frozen in liquid nitrogen and stored at −80 °C for molecular analyses. All procedures were approved by the French Ministry of Research and the local ethics committee (CEEA - 006) and complied with EU Directive 2010/63/EU.

### 4.2 Histology and tissue staining

Adipose and liver tissues were fixed in 10% PBS-buffered formalin for at least 24 h, paraffin-embedded, and sectioned at 5 μm. After deparaffinization and rehydration with specific commercial solutions (Impath), sections were stained with with hematoxylin and eosin for morphology, picrosirius red to assess fibrosis and periodic acid–Schiff to evaluate glycogen content. Whole-slide liver images were acquired using an Aperio digital slide scanner (Scanscope CS2 System, Aperio Technologies, Vista, CA, USA), providing high-resolution images (maximum scanning area capacity of 120,000 × 50,000 pixels at 0.5 μm/pixel, magnification × 20). Adipocyte diameter (VAT, SAT), steatosis and fibrosis areas (liver) were quantified using dedicated ImageJ plugins.

### 4.3 RNA extraction and qPCR

RNA was extracted from liquid nitrogen–frozen tissues. For VAT, RNA was isolated using QIAzol and purified with the RNeasy Microkit (Qiagen), including DNase I treatment. Liver and muscle RNA were extracted using the Maxwell® RSC simplyRNA Tissue Kit and instrument (Promega). RNA quantity and purity were assessed via NanoDrop ND-2000 (Thermo Fisher). cDNA was synthesized from 1 μg RNA using SuperScript™ II (Invitrogen) and random hexamers, then purified with the Qiaquick PCR kit (Qiagen). qPCR was performed with 3 ng cDNA, Maxima™ SYBR Green Master Mix (Thermo Fisher), and 0.3 μM primers using a CFX Opus 384 system (Bio-Rad). Cycling conditions were 95 °C for 10 min, then 40 cycles of 95 °C for 15 s and 60 °C for 30 s. Specificity was confirmed by melting curve analysis. Gene expression was calculated via the 2^−ΔΔCq^ method, normalized to 18S and 36B4, and analyzed with CFX Maestro (Bio-Rad). Primer sequences are available upon request.

### 4.4 AKT and AMPK signaling

Insulin and AMPK signaling were assessed in mouse tissues collected 15 min after i.p. insulin injection. VAT, SAT, and skeletal muscle (quadriceps) were harvested at this time point. Tissue homogenization was performed on 20 mg of snap-frozen liver or muscle tissue and 100 mg of snap-frozen VAT using CK14 soft tissue beads and the Precellys homogenizer, according to the manufacturer's instructions. Protein lysates (15 μg) were subjected to SDS–PAGE and Western blotting, as previously described^11^. Primary antibodies used are phospho-ACC (Ser79, #11818), total ACC (pan, #3676), phospho-Akt (Ser473, #4060), total Akt (pan, #4691), phospho-AMPKα (Thr172, #2535) and total AMPKα (#2532) (Cell Signaling Technology), FAS (kind gift from Dr I. Dugail, France), GAPDH (#ABC-AC002-100, ABclonal), and β-actin (#A5316, Sigma–Aldrich). IRDye secondary antibodies and an Odyssey CLx imaging system (LI-COR) were use for detection and quantification was performed using Image Studio. Representative blots include samples from at least three independent animals per group.

### 4.5 Hepatic lipid and glycogen quantification

Hepatic lipids were extracted from snap-frozen liver samples using a modified Bligh and Dyer method [23]. Triglycerides and total cholesterol were quantified using enzymatic kits (Roche Diagnostics and DiaSys) and expressed as total liver content. Total liver glycogen content was quantified by colorimetric assay following the manufacturer's protocol (Abcam).

### 4.6 Ceramide analysis by mass spectrometry

Ceramides were quantified from 10 μL of plasma using targeted lipid liquid chromatography–tandem mass spectrometry (LC-MS/MS), as previously described [24].

### 4.7 FGF21 measurment

Circulating FGF21 concentrations was measured using commercial ELISA kits designed for mouse measurments (R&D systems) from plasma samples according to the manufacturer's protocol.

### 4.8 Quantification and statistical analysis

Data were analyzed with GraphPad Prism and presented as dot plots of independent experiments. Statistical tests used are specified in figure legends. Significance was set at p ≤ 0.05, indicated as ∗p ≤ 0.05, ∗∗p ≤ 0.01, ∗∗∗p ≤ 0.005, and ∗∗∗∗p ≤ 0.001.

## CRediT authorship contribution statement

**Alexia Blandin:** Writing – review & editing, Methodology, Formal analysis. **Margot Voisin:** Writing – review & editing, Methodology, Formal analysis. **Josy Froger:** Writing – review & editing, Methodology, Formal analysis. **Maëlle Lachat:** Methodology, Formal analysis. **Grégory Hilairet:** Methodology, Formal analysis. **Katarzyna Polak:** Methodology, Formal analysis. **Julie Magusto:** Methodology, Formal analysis. **Lisa Meslier:** Methodology, Formal analysis. **Mikaël Croyal:** Methodology, Formal analysis. **Mathilde Gourdel:** Methodology, Formal analysis. **Quentin Massiquot:** Methodology, Formal analysis. **Julien Chaigneau:** Methodology, Formal analysis. **Maxime Carpentier:** Methodology, Formal analysis. **Lionel Fizanne:** Methodology, Formal analysis. **Xavier Prieur:** Writing – review & editing, Methodology. **Cédric Le May:** Writing – review & editing, Methodology. **Jérôme Boursier:** Writing – review & editing. **Bertrand Cariou:** Writing – review & editing, Conceptualization. **Robert Mamoun:** Writing – review & editing, Methodology, Conceptualization. **Bernadette Trentin:** Writing – review & editing, Methodology, Conceptualization. **Soazig Le Lay:** Writing – review & editing, Writing – original draft, Validation, Supervision, Software, Resources, Project administration, Methodology, Investigation, Funding acquisition, Formal analysis, Conceptualization.

## Financial support

The authors were supported the 10.13039/501100001665French National Research Agency (ANR-22-CE18-0026-02 EVADIPO). SLL is granted by Genavie, FHU GO NASH, Société Francophone du Diabète and the FFRD (sponsored by Fédération Française des Diabétiques, Abbott, 10.13039/100004325AstraZeneca, Eli Lilly, Merck Sharp & Dohme, and 10.13039/501100004191Novo Nordisk).

## Declaration of competing interest

The authors declare the following financial interests/personal relationships which may be considered as potential competing interests:Soazig Le Lay and Ciloa company reports financial support was provided by French National Research Agency - ANR-22-CE18-0026-02 EVADIPO). Soazig Le Lay reports financial support was provided by Genavie. Soazig Le Lay reports financial support was provided by FHU GO NASH. Soazig Le Lay reports financial support was provided by Société Francophone du Diabète and the FFRD (sponsored by Fédération Française des Diabétiques, Abbott, AstraZeneca, Eli Lilly, Merck Sharp & Dohme, and Novo Nordisk). Robert Mamoun reports a relationship with Ciloa SAS that includes: board membership and employment. Bernadette Trentin reports a relationship with Ciloa SAS that includes: board membership and employment. Blandin Alexia reports a relationship with Ciloa SAS that includes: employment. Lachat Maelle reports a relationship with Ciloa SAS that includes: employment. Polak Katarzyna reports a relationship with Ciloa SAS that includes: employment. TRENTIN Bernadette, POLAK Katarzyna, MAMOUN Robert has patent # WO2023104822A1 issued to Ciloa SAS. If there are other authors, they declare that they have no known competing financial interests or personal relationships that could have appeared to influence the work reported in this paper.

## Data Availability

Data will be made available on request.
