# Supplementary material for: Extracellular vesicles carrying surface-anchored adiponectin prevent obesity-related metabolic complications by enhancing insulin sensitivity
Source: Mol Metab. 2026 Apr 1;107:102361. doi: 10.1016/j.molmet.2026.102361 (PMC13092689; doi:10.1016/j.molmet.2026.102361)
Supplement: Multimedia component 1 [file mmc1.docx]

**Table S1**

**Table S1: Blood biochemical profiles in male and female mice treated with bioengineered EVs.**

Data are presented as mean ± SEM for male (♂) and female (♀) mice treated with bioengineered EVs (25 ng Adpn-equivalent). The number of animals analyzed per group is indicated (*n*). Statistical differences were determined using one-way ANOVA followed by **Tukey’s multiple comparisons test.** * indicates p < 0.05 versus Vehicle, while ^###^ indicates p < 0.005 versus EV^CTL^. *ND*, not determined.
