# Supplementary material for: Extracellular vesicles carrying surface-anchored adiponectin prevent obesity-related metabolic complications by enhancing insulin sensitivity
Source: Mol Metab. 2026 Apr 1;107:102361. doi: 10.1016/j.molmet.2026.102361 (PMC13092689; doi:10.1016/j.molmet.2026.102361)
Supplement: Multimedia component 2 [file mmc2.docx]

**Figure S1. Design and validation of bioengineered EV-anchored Adpn.**

**(A)** Schematic representation of Adpn molecular construct used to establish a stable cell line expressing EV-anchored Adpn (EV^PP-Adpn^) via the fusion of adiponectin gene at its N-terminus to a sequence encoding a transmembrane domain (TM) and a pilot peptide (PP). Control EVs (EV^CTL^) lacking Adpn were produced from mock-transfected HEK293T cells.

**(B-C)** Enrichment of EV markers in bioengineered EVs. The presence of the EV marker proteins Alix and Syntenin-1 was assessed by Western blot **(B)**. Presence of the EV marker CD81 in EVs assessed by ELISA **(C)**. Standard EVs were used as positive EV marker controls (C+). Representative blots for both EV^CTL^ and EV^PP-Adpn^ are shown.

**(D-F)** Size distribution curves **(D)**, EV mean size **(E),** and EV concentration **(F)** of bioengineered EVs, as measured using a NanoFCM instrument (Nano-FC). EV concentrations were additionally assessed by NTA (ZetaView) in panel F. Dot plots represent independent production batches for each EV type. In Panel F, results are presented as mean ± sem (n=4 for EV^CTL^, n=3 for EV^PP-Adpn^).

**(G)** Adpn content was quantified by ELISA and expressed as ng Adpn per µg EV protein. Dot plots represent independent production lots per EV type analyzed.

**(H)** Western-blot analysis of EV-anchored Adpn (EV^PP-Adpn^) under reducing and non-reducing **(**conditions, demonstrating the presence of Adpn and its assembly under high-molecular-weight proteo-oligomeric forms.

**(I)** Corresponding injected EV numbers for EV^PP-Adpn^ and EV^CTRL^ at the 25-ng Adpn-equivalent dose, as quantified by Nano-Flow cytometry (NanoFC) or by NTA (ZetaView). Results are presented as mean ± sem (n=3 for EV^CTL^, n=2 for EV^PP-Adpn^).
