# Supplementary material for: Extracellular vesicles carrying surface-anchored adiponectin prevent obesity-related metabolic complications by enhancing insulin sensitivity
Source: Mol Metab. 2026 Apr 1;107:102361. doi: 10.1016/j.molmet.2026.102361 (PMC13092689; doi:10.1016/j.molmet.2026.102361)
Supplement: Multimedia component 3 [file mmc3.docx]

**Figure S2. Metabolic phenotyping of EV^CTL^, EV^PP-Adpn^ or Vehicle injected HFD-fed mice**

**(A)** Tissue weight (in g) at sacrifice is presented for liver, VAT, SAT, BAT, pancreas, spleen and muscle following EV injections in males (left panel) or in females (right panel). Floating bars (min to max) with line at mean are presented for each condition.

**(B-C)** Random-fed glycemia measured throughout the experiment, n=16-18 for males (top); n=9-10 for females (bottom) **(B)** and fasting glycemia measured at sacrifice **(C)** in males (top) and females (bottom).

**(D)** Insulin levels measured at baseline (T0) and 15 minutes post-glucose injection (T15) during GTT in males (top) and females (bottom). Five animals per group were analyzed.

**(E-H)** Representative pancreatic sections stained for insulin showing comparable β-cell mass across treatment groups in male mice **(E)**. Quantification of β-cell fraction (%) **(F)**, mean islet size (µm²) **(G)**, and islet density (n/mm²) **(H)** are shown. Scale bar, 2 nm.

**(I-J)** Histological analysis of SAT sections in male (upper panels) and female (lower panels) mice following the EV injection protocol showed no significant changes in adipocyte size distribution **(I)** or mean adipocyte area **(J)** across treatment groups.
