# Supplementary material for: Extracellular vesicles carrying surface-anchored adiponectin prevent obesity-related metabolic complications by enhancing insulin sensitivity
Source: Mol Metab. 2026 Apr 1;107:102361. doi: 10.1016/j.molmet.2026.102361 (PMC13092689; doi:10.1016/j.molmet.2026.102361)
Supplement: Multimedia component 4 [file mmc4.docx]

**Supplementary Material**

**Extracellular vesicles carrying surface-anchored adiponectin prevent obesity-related metabolic complications by enhancing insulin sensitivity**

**Supplementary Methods**

**Gene synthesis and molecular cloning**

Gene encoding adiponectin (accession number: NM_001177800.2), were codon optimized for human cells to generate Adiponectin gene. A PP-Adpn chimeric construct was generated encoding the adiponectin gene (Adpn) fused at its N-terminus to a sequence encoding transmembrane domain (TM) and to the sequence encoding a pilot peptide (PP, Patent WO2023104822A1, [1]). This construct was subcloned into a eukaryotic expression plasmid that also carries a zeocin resistance gene.

**Cell culture and EV production**

HEK293T were cultured in DMEM supplemented with 5% heat inactivated fetal bovine serum (iFBS), 2 mM GlutaMAX and 5 µg/mL of gentamicin at 37 °C in a 5% CO2 humidified incubator. HEK293T cells were transfected with adiponectin DNA using PEI. Zeocin (Invivogen) selection pressure was applied in order to establish stable cell lines. Stably transfected HEK293T cells expressing the PP-Adpn chimeric construct were cultured in cell chambers of 10 trays in complete medium and used for EV^PP-Adpn^ production. Twenty-four hours after, cultures were fed with an EV-free medium and incubated for a further 48 hours. Mock-transfected HEK293T cells were used for the production of control EVs (EV^CTL^) lacking adiponectin.

**EV purification**

Conditioned cell culture medium was harvested and the EV isolation was performed as previously described [2]. Briefly, cell culture supernatant was clarified by two consecutive centrifugations: 10 minutes at 1300 rpm and 15 minutes at 4000 rpm, both at 4 °C, followed by filtration through 0.22 µm membrane filters. The supernatant was then concentrated by ultra-filtration/diafiltration and purified by Size Exclusion-based Chromatography. Fractions containing EV CD81 marker were identified by ELISA and pooled.

**EV size distribution and particle number**

EV size distribution and absolute size distribution were obtained by nanoflow cytometry (Nano-FC) using a NanoAnalyzer instrument (NanoFCM). EV concentrations were also measured by NTA using the ZetaView system to provide an additional quantification of particle numbers, as the two systems rely on different measurement principles. The results were validated through at least three replicates.

**SDS-PAGE, Western blotting and antibodies**

Protein concentrations of EV batches were determined using the BCA assay (Thermo Scientific). For the identification of EV markers (Alix and Syntenin-1) by SDS-PAGE, 5 μg of pure EVs were lysed in denaturing buffer and heated for 10 min at 95°C. For the detection of adiponectin, 1.25 μg of pure EVs were lysed in denaturing buffer and heated for 10 min at 95°C. Identification of adiponectin multimeric forms was performed in non-reducing and unheated conditions. EV preparations were separated by SDS-PAGE on a 4-15% gradient polyacrylamide gel (Bio-Rad) and proteins were subsequently transferred onto PVDF membrane. The immunodetection of proteins was performed with primary antibodies recognizing specifically either Alix (Proteintech), Adiponectin (Genetex) or Syntenin-1 (Fisher Scientific) proteins. Membranes were then incubated with the corresponding secondary Horseradish Peroxidase (HRP)-conjugated antibodies (donkey anti-goat HRP, donkey anti-mouse HRP or donkey anti-rabbit HRP, Jackson ImmunoResearch). The signals were detected using enhanced chemiluminescence detection.

**Anti-CD81 ELISA**

Serial dilutions of pure EVs (from 1 μg to 1 ng) were coated onto a 96-well ELISA plate overnight at 4°C. After saturation with 3% BSA in PBS during 1 h at 37°C, anti-CD81 was added and incubated for 2 h at 37°C. Then, the plate was washed three times and incubated with the corresponding secondary HRP-conjugated antibody for 1 h at 37°C. After washing 5 times, 3, 3', 5, 5' – Tetramethylbenzidine (TMB), the chromogenic peroxidase substrate, was added and the plate was incubated under dark for 30 min at room temperature. Sulfuric acid was added to stop the reaction. Optical density (OD) was measured at 450 nm using a CLARIOstar Plus plate reader (BMG Labtech).

**Quantitative anti-Adiponectin ELISA**

The adiponectin concentration was measured using commercial ELISA kits (Human Adiponectin DuoSet ELISA, R&D systems) designed to measure full-length human adiponectin (Adpn) levels according to the manufacturer’s protocol. 3 ng of Adpn EVs were lysed and diluted in 1X diluent reagent provided in the kit. The capture and HRP-conjugated detection antibodies as well as the calibrator provided in the total adiponectin ELISA kit were used. After substrate solution incubation, the optical density at 450 nm was read with a CLARIOstar Plus plate reader and corrected by the optical density read at 570 nm. Adpn concentrations were calculated from a four-parameter logistic (4-PL) standard curve fitting.

**Biochemical analysis of circulating metabolic markers**

Serum metabolic and hepatic parameters were measured from 100 µL of randomly fed mouse serum using a Cobas Pro analyzer (Roche Diagnostics). The following analytes were quantified using dedicated reagents: total protein, albumin, glucose, triglycerides, total cholesterol, HDL cholesterol, LDL cholesterol, alanine aminotransferase (ALT), aspartate aminotransferase (AST), and alkaline phosphatase (ALP). All measurements were performed according to the manufacturer’s protocols, with automatic internal quality control checks ensuring analytical reliability and reproducibility.

Circulating free fatty acids were measured using Non-Esterified Fatty Acid (NEFA) Assay Kit (Fujifilm) following the the manufacturer’s protocol.

Plasma insulin during GTT was quantified by ELISA (Crystal Chem) from heparinized blood.

**Quality control of EV batches**

EV^PP-Adpn^ stability was confirmed over six months, as evidenced by consistent particle concentration and size distribution (NanoFCM), preserved EV integrity (CD81 ELISA), maintenance of adiponectin multimerization (non-reducing Western blot), and stable total adiponectin content (quantitative ELISA).

**Supplemental references**

[1] Mamoun, R., Trentin, B., Polak, K., 2023. . Chimeric adiponectin polypeptides, extracellular vesicle comprising the same, and uses thereof., World Intellectual Property Organization (WIPO). CILOA (FR), International.

[2] Desplantes, R., Leveque, C., Muller, B., Lotierzo, M., Ferracci, G., Popoff, M., et al., 2017. Affinity biosensors using recombinant native membrane proteins displayed on exosomes: application to botulinum neurotoxin B receptor. Sci Rep 7(1):1032.
